# Gender Differences Between the Phenotype of Short Stature and the Risk of Diabetes Mellitus in Chinese Adults: A Population-Based Cohort Study

**DOI:** 10.3389/fendo.2022.869225

**Published:** 2022-04-05

**Authors:** Wei Song, Yaqin Hu, Jiao Yuan, Ying Wei, Zongyou Cheng, Jingdong Liu, Jixiong Xu, Xiaoyu Wang

**Affiliations:** ^1^ Department of Endocrinology, Jiangxi Provincial People’s Hospital, The First Affiliated Hospital of Nanchang Medical College, Nanchang, China; ^2^ Department of Pharmacy, Nanchang First Hospital, Nanchang, China; ^3^ Department of Endocrinology, The First Affiliated Hospital of Nanchang University, Nanchang, China

**Keywords:** diabetes mellitus, gender differences, general population, height, short stature, Chinese, cohort study

## Abstract

**Objective:**

Previous studies have shown that there are significant regional and gender differences in the association between the phenotype of short stature and diabetes mellitus (DM). The purpose of this study was to investigate the gender difference between the phenotype of short stature and the risk of DM in the Chinese population.

**Methods:**

The sample included 116,661 adults from 32 locations of 11 cities in China, of which the average height of men and women was 171.65 and 160.06 cm, respectively. Investigators retrospectively reviewed annual physical examination results for follow-up observations and set confirmed DM events as the outcome of interest. Multivariate Cox regression, restricted cubic spline, and piecewise regression models were used to check the association between height and DM risk.

**Results:**

During an average observation period of 3.1 years, there were 2,681 of 116,661 participants who developed new-onset DM, with a male to female ratio of 2.4 to 1. After full adjustment for confounders, we confirmed that there was a significant negative correlation between height and DM risk in Chinese women (HR per 10 cm increase: 0.85, 95% CI: 0.74–0.98), but not in men (HR per 10 cm increase: 1.16, 95% CI: 0.98–1.14). Additionally, through restricted cubic spline and piecewise regression analysis, we determined that the height of 157–158 cm may be the critical point for short stature used to assess the risk of DM in Chinese women.

**Conclusions:**

In the Chinese population, female short stature phenotype is related to increased DM risk, among which 157–158 cm may be the saturation effect point of female short stature for predicting DM risk.

## Introduction

Diabetes mellitus (DM) is a chronic non-infectious disease characterized by elevated glucose levels due to disturbances in glucose metabolism, 90% of which are type 2 DM, which is an important cause of physical disability and death (1, 2). With the global obesity epidemic, the aging population, and the great changes in lifestyle and dietary patterns, the prevalence of DM in China has doubled in the past 30 years (1995–1999: 4.5%; 2010–2014: 8.35%; 2019: 116 million) (3–7). At present, China has become the center of the global DM pandemic and has the largest number of DM patients in the world (7).

In the past few decades, a large number of studies have found that patients with DM are often accompanied by some special body phenotypes, such as obesity phenotype, high waist circumference phenotype, hypertriglyceridemic waist phenotype, and short stature phenotype (3, 8–10). In other words, these special body phenotypes can help us assess DM risk. The phenotype of short stature has been shown to be closely related to the increased risk of DM in previous studies, but there is still some debate about this association between different regions and between genders (11–16). In the existing longitudinal correlation studies involving both men and women, the findings of England and Germany have supported that only the height of men was negatively correlated with the risk of DM (11, 12); results from Norway and Iran have supported a negative association between height and DM risk only in women (13, 14); South Korea’s research has shown that there was a negative correlation between height and DM risk in both sexes (15), while the United States study has found no association between height and DM (16). Although the results of these studies were not identical, it further indicated that there are significant regional and gender differences in the association between height and DM. China, as a hardest-hit area of DM disease burden, currently has very limited data on the correlation between height and DM, so it is necessary to determine the gender difference between height and DM risk and the appropriate risk threshold or saturation point in the Chinese population as soon as possible. To address this issue, the present study conducted an in-depth analysis of national physical examination data from Rich Healthcare Group in China to identify gender differences in height and DM risk among Chinese adults and to determine an appropriate height threshold or saturation point for predicting future DM risk.

## Methods

### Data Sources and Study Design

In this study, we conducted a secondary analysis of the retrospective cohort study based on the national physical examination data of China Rich Healthcare Group. The original data have been shared to the public database (www.Datadryad.org) by Chen et al. (17).

**Figure 1:**
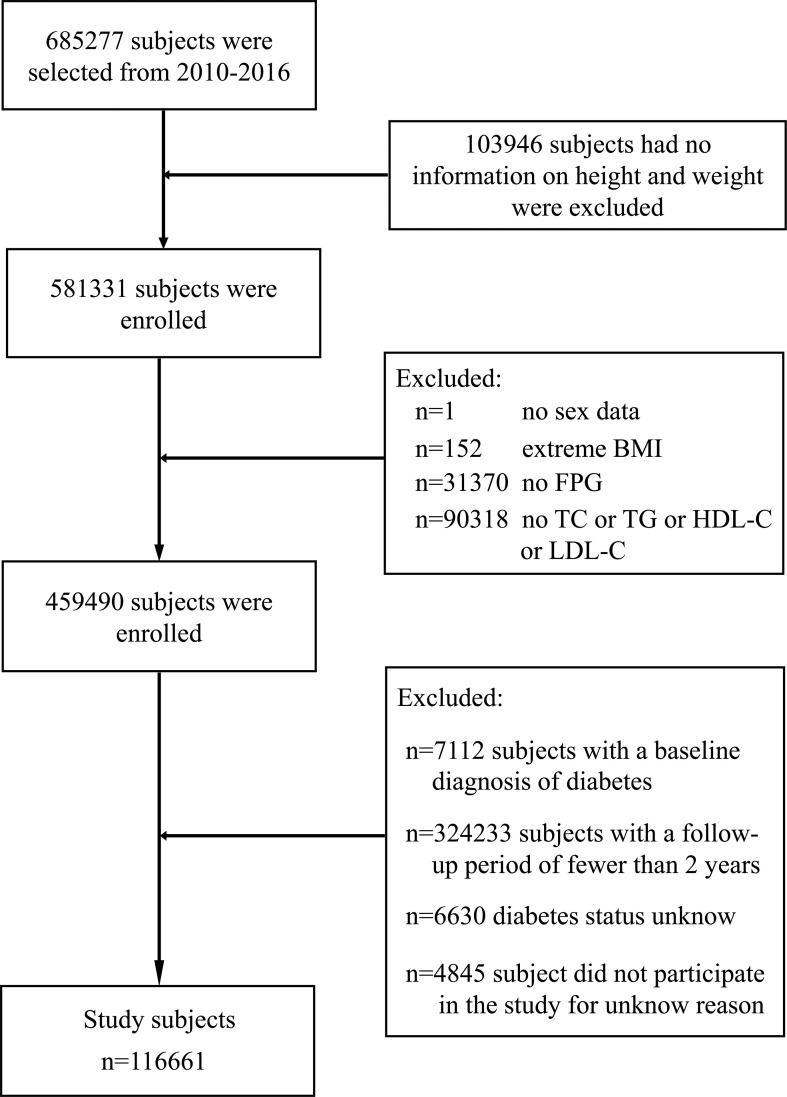
Flow diagram of subjects included in the cohort study.

The study design of the retrospective cohort has been described in detail in previous studies (18). In short, the current study cohort was from adults who underwent health screening in China Rich Healthcare Group from 2010 to 2016 (*n* = 685,277); considering that these participants were screened at least twice during this period, therefore, a retrospective analysis can be conducted based on the research data of this population. In previous studies by Chen et al., they retrospectively analyzed the association between body mass index (BMI) and DM risk (18). Given the chronic course of DM, they excluded participants from the previous study who were followed for less than 2 years (*n* = 324,233). Moreover, for study purposes, they also excluded participants with incomplete or extreme baseline BMI (BMI > 55 or <15 kg/m^2^; *n* = 152); participants with no gender, height, weight, or fasting plasma glucose (FPG) information at baseline (n=135,317); participants with diagnosed DM at baseline (n=7,112); and participants with unknown DM status during follow-up (*n* = 6,630). Ultimately, Chen et al. included 211,833 participants who met the criteria for their analysis. Based on the data used by Chen et al., the current study further excluded participants with loss of baseline lipid parameters (*n* = 95,172) and finally included 116,661 participants (**Figure 1**). These people come from 32 locations of 11 cities in China, accounting for 7/34 of China’s provincial administrative regions and 8.26/100,000 of China’s total population. The Ethics Committee of Jiangxi Provincial People’s Hospital approved the research protocol (ethical review no. 2021-067). Also, considering that the identification information of the participants in the current study was canceled, the Institutional Ethics Committee of Jiangxi Provincial People’s Hospital waived the informed consent of the participants.

### Health Examination and Laboratory Measurement

As mentioned earlier (18), the trained medical staff recorded the baseline clinical data of the participants during the physical examination through a standard questionnaire, including age, height, blood pressure, gender, family history of DM, weight, and smoking and drinking status. The medical staff used an automatic scale to measure the height and weight of the participants, during which the participants took off their shoes and wore only light clothes. Blood pressure was measured using a standard mercury sphygmomanometer. BMI is calculated from height and weight.

Participants’ venous blood samples were obtained at least 10 h after fasting at each physical examination. High-density lipoprotein cholesterol (HDL-C), triglyceride (TG), FPG, total cholesterol (TC), low-density lipoprotein cholesterol (LDL-C), aspartate aminotransferase (AST), alanine aminotransferase (ALT), creatinine (Cr), and blood urea nitrogen (BUN) were measured by standard experimental methods on an automatic analyzer (Beckman 5800).

### Identification of DM

Participants were followed up on the basis of annual health checkups until December 2016. According to the American Diabetes Association’s diagnostic criteria for DM, DM was defined as measured FPG ≥7.00 mmol/L or self-reported diagnosis of DM during follow-up (19).

### Statistical Analysis

R language software (version 3.4.3) and Empower(R) (version 2.20) software were used to analyze the data of this study. All baseline data were expressed as mean or median or percentage, respectively, where appropriate. One-way ANOVA or *t*-test or Kruskal–Wallis *H* test was used to compare the mean (median) of continuous variables, and the chi-square test was used to compare categorical variables between groups. All *P*-values were bilateral, and *P <*0.05 was the significant standard.

In multivariate Cox regression analysis, we ran three models with DM events as endpoints, identified relevant confounding factors based on epidemiology, and recorded the hazard ratio (HR) and 95% confidence interval (CI) related to height and DM events (20). Before running the multivariate Cox regression model, we checked for collinearity between all covariables (21), among which weight and TC were excluded from the model due to variance inflation factor greater than 5. Model 1 was adjusted for age, BMI, FPG, and DM family history. Model 2 further considered the effects of blood pressure, smoking, and drinking on DM on the basis of model 1. Model 3 was further adjusted for BUN, Cr, TG, LDL-C, and HDL-C. For the selection of the best model, we designated model 3 which has adjusted all non-collinear variables as the final model after the epidemiological and statistical screening.

Restricted cubic splines (RCS: nested in Cox regression analysis) with four knots were used to fit the shape of the dose–response correlation between height and the risk of DM (22, 23). By visually examining the shape of the curve, we selected the critical point when HR changes from larger or smaller to 1 to serve as the height threshold point or saturation effect point (if any) used to assess the DM risk. If the potential threshold or saturation effect point between height and DM risk was found by RCS, we will further use the piecewise regression model to calculate the threshold or saturation effect point by using a recursive algorithm (24).

We also examined the HR and 95% CI of height and the risk of DM in different age and BMI populations, where the cutoff point of BMI was based on the classification standard recommended by the Chinese Obesity Working Group (25) and the cutoff point of age was based on the age classification standard of the World Health Organization in 2000. Likelihood ratio tests were used to compare whether there were differences in height-related DM risk among different age and BMI groups.

## Results

### Study of the Baseline Characteristics of the Population

A total of 116,661 participants without DM at baseline were included in the current study, with a male to female ratio of 1.16:1 and a mean age of 44 and 43 years, respectively. In consideration of the significant gender differences in previous similar studies (11–16), the baseline characteristics of men and women grouped by independent and dependent variables were summarized in this study.

**Table 1** presents the quartiles of height, showing the baseline characteristics of men and women in different height categories. In both sexes, with the increase of the quartile of height, the weight and Cr levels increased gradually; in contrast, BMI, age, blood glucose, blood pressure, blood lipid, and AST and BUN levels decreased gradually. The gender differences were mainly reflected in ALT levels and smoking and drinking. In men, the level of ALT gradually increased with the increase of height, while in women, the trend of ALT level and height seemed to be the opposite; additionally, the basic ALT level of men was higher than that of women.

**Table 1:** Baseline characteristics of four groups in men and women.

	Height quartiles				*P*-value
	Q1	Q2	Q3	Q4	
**Men**					
**Age, years**	55.00 (41.00–67.00)	48.00 (37.00–60.00)	43.00 (35.00–55.00)	38.00 (33.00–49.00)	<0.001
**Weight, kg**	60.50 (7.87)	65.39 (8.26)	69.58 (8.97)	75.67 (10.67)	<0.001
**BMI, kg/m^2^**	24.50 (3.14)	24.52 (3.06)	24.38 (3.11)	24.23 (3.24)	<0.001
**SBP, mmHg**	128.60 (19.33)	125.07 (17.02)	123.27 (15.90)	121.90 (14.88)	<0.001
**DBP, mmHg**	78.27 (11.70)	77.64 (11.01)	77.18 (10.84)	76.50 (10.56)	<0.001
**FPG, mmol/L**	5.12 (0.68)	5.07 (0.64)	5.01 (0.63)	4.97 (0.62)	<0.001
**TC, mmol/L**	4.85 (4.23–5.47)	4.80 (4.20–5.40)	4.76 (4.20–5.37)	4.70 (4.16–5.30)	<0.001
**TG, mmol/L**	1.29 (0.90–1.89)	1.34 (0.94–2.00)	1.33 (0.92–1.97)	1.31 (0.91–1.94)	0.002
**HDL-C, mmol/L**	1.30 (1.11–1.52)	1.29 (1.10–1.49)	1.28 (1.10–1.47)	1.26 (1.09–1.45)	<0.001
**LDL-C, mmol/L**	2.78 (2.35–3.26)	2.75 (2.34–3.22)	2.74 (2.33–3.20)	2.72 (2.31–3.17)	<0.001
**ALT, U/L**	21.80 (15.90–31.00)	22.70 (16.50–33.00)	23.10 (17.00–34.00)	23.40 (16.90–34.80)	<0.001
**AST, U/L**	25.00 (21.45–31.00)	24.10 (20.90–29.00)	24.00 (20.00–29.00)	23.00 (19.70–28.00)	<0.001
**BUN, mmol/L**	4.90 (4.20–5.89)	4.90 (4.20–5.72)	4.82 (4.11–5.62)	4.79 (4.10–5.57)	<0.001
**Cr, μmol/L**	78.40 (70.50–87.00)	79.00 (71.50–87.25)	79.20 (72.00–87.00)	80.30 (73.10–88.00)	<0.001
**Family history of diabetes**	14 (0.85%)	110 (1.26%)	347 (1.60%)	557 (1.81%)	<0.001
**Smoking status**					<0.001
** No**	161 (9.82%)	921 (10.58%)	2,419 (11.19%)	3,140 (10.20%)	
** Former**	31 (1.89%)	178 (2.04%)	439 (2.03%)	667 (2.17%)	
** Current**	409 (24.94%)	2,044 (23.48%)	4,605 (21.30%)	6,206 (20.15%)	
** Not recorded**	1,039 (63.35%)	5,562 (63.89%)	14,158 (65.48%)	20,780 (67.48%)	
**Drinking status**					<0.001
** No**	30 (1.83%)	135 (1.55%)	308 (1.42%)	382 (1.24%)	
** Former**	113 (6.89%)	668 (7.67%)	1,806 (8.35%)	2,628 (8.53%)	
** Current**	458 (27.93%)	2,340 (26.88%)	5,349 (24.74%)	7,003 (22.74%)	
** Not recorded**	1,039 (63.35%)	5,562 (63.89%)	14,158 (65.48%)	20,780 (67.48%)	
**Women**					
**Age, years**	44.00 (35.00–57.00)	39.00 (33.00–49.00)	37.00 (32.00–46.00)	35.00 (31.00–42.00)	<0.001
**Weight, kg**	54.68 (7.58)	57.78 (7.87)	60.81 (8.22)	64.69 (9.09)	<0.001
**BMI, kg/m^2^**	22.67 (3.12)	21.90 (2.97)	21.56 (2.89)	21.39 (2.95)	<0.001
**SBP, mmHg**	117.47 (18.14)	113.65 (15.59)	112.52 (14.29)	112.54 (13.51)	<0.001
**DBP, mmHg**	72.15 (10.88)	71.02 (10.16)	70.82 (10.02)	70.95 (9.83)	<0.001
**FPG, mmol/L**	4.92 (0.58)	4.85 (0.56)	4.84 (0.55)	4.81 (0.55)	<0.001
**TC, mmol/L**	4.75 (4.19–5.41)	4.61 (4.10–5.23)	4.55 (4.05–5.17)	4.52 (4.01–5.08)	<0.001
**TG, mmol/L**	0.96 (0.69–1.40)	0.87 (0.63–1.23)	0.82 (0.61–1.17)	0.80 (0.61–1.14)	<0.001
**HDL-C, mmol/L**	1.45 (1.26–1.65)	1.45 (1.26–1.65)	1.46 (1.27–1.68)	1.46 (1.28–1.67)	<0.001
**LDL-C, mmol/L**	2.71 (2.29–3.21)	2.63 (2.23–3.08)	2.59 (2.21–3.04)	2.56 (2.18–3.00)	<0.001
**ALT, U/L**	14.60 (11.20–20.00)	13.80 (10.90–18.70)	13.20 (10.50–17.60)	13.00 (10.20–17.80)	<0.001
**AST, U/L**	21.00 (18.00–25.00)	20.00 (17.00–23.60)	19.00 (16.50–22.80)	18.40 (16.00–22.00)	<0.001
**BUN, mmol/L**	4.32 (3.63–5.15)	4.20 (3.55–5.00)	4.13 (3.50–4.90)	4.02 (3.43–4.79)	<0.001
**Cr, μmol/L**	57.00 (51.40–63.00)	57.40 (52.00–63.50)	58.20 (52.80–64.20)	58.90 (53.58–64.62)	<0.001
**Family history of diabetes**	740 (2.90%)	602 (3.05%)	233 (3.08%)	31 (2.88%)	0.763
**Smoking status**					<0.001
** No**	8 (0.03%)	7 (0.04%)	1 (0.01%)	1 (0.09%)	
** Former**	5 (0.02%)	5 (0.03%)	1 (0.01%)	0 (0.00%)	
** Current**	5,745 (22.52%)	3,996 (20.24%)	1,448 (19.13%)	196 (18.22%)	
** Not recorded**	19,751 (77.43%)	15,738 (79.70%)	6,121 (80.85%)	879 (81.69%)	
**Drinking status**					<0.001
** No**	9 (0.04%)	4 (0.02%)	3 (0.04%)	1 (0.09%)	
** Former**	149 (0.58%)	111 (0.56%)	43 (0.57%)	6 (0.56%)	
** Current**	5,600 (21.95%)	3,893 (19.72%)	1,404 (18.54%)	190 (17.66%)	
** Not recorded**	19,751 (77.43%)	15,738 (79.70%)	6,121 (80.85%)	879 (81.69%)	

Values were expressed as mean (SD) or medians (quartile interval) or n (%).

BMI, body mass index; SBP, systolic blood pressure; DBP, diastolic blood pressure; FPG, fasting plasma glucose; TG, triglyceride; TC, total cholesterol; LDL-C, low-density lipid cholesterol; BUN, blood urea nitrogen; Cr, creatinine; ALT, alanine aminotransferase; AST, aspartate aminotransferase.

**Table 2** summarizes the baseline characteristics of both sexes according to the presence or absence of new-onset DM during the follow-up. During an average follow-up period of 3.1 years, a total of 2,681 participants developed new-onset DM (518 people with a self-reported diagnosis of DM), with a male to female ratio of 2.4 to 1. Regardless of gender, participants ultimately diagnosed with DM had higher levels of TC, ALT, FPG, BUN, LDL-C, TG, AST, weight, BMI, age, SBP, and DBP at baseline. Compared with men, women had higher levels of age, SBP, and blood lipids and lower levels of height, weight, BMI, DBP, FPG, BUN, Cr, and liver enzymes.

**Table 2:** Characteristics of the study participants with and without DM.

	Men				Women			
	Non-DM	DM	*P*-value	Cohen’s *d*	Non-DM	DM	*P*-value	Cohen’s *d*
**No. of participants**	60,871	1,888			53,109	793		
**Age, years**	41.00 (34.00–53.00)	56.00 (47.00–64.00)	<0.001	0.80	41.00 (34.00–52.00)	60.00 (51.00–67.00)	<0.001	1.00
**Height, cm**	171.69 (6.23)	170.31 (6.47)	<0.001	0.22	160.09 (5.65)	157.53 (5.75)	<0.001	0.45
**Weight, kg**	71.59 (10.54)	76.91 (11.85)	<0.001	0.50	56.79 (8.14)	62.17 (9.46)	<0.001	0.66
**BMI, kg/m^2^**	24.26 (3.14)	26.45 (3.30)	<0.001	0.70	22.16 (3.04)	25.04 (3.52)	<0.001	0.95
**SBP, mmHg**	122.72 (15.58)	131.54 (18.21)	<0.001	0.56	115.01 (16.58)	132.95 (20.03)	<0.001	1.08
**DBP, mmHg**	76.80 (10.70)	81.52 (11.65)	<0.001	0.44	71.42 (10.43)	78.32 (12.26)	<0.001	0.66
**TC, mmol/L**	4.73 (4.19–5.33)	4.94 (4.34–5.60)	<0.001	0.23	4.66 (4.11–5.30)	5.20 (4.48–5.90)	<0.001	0.52
**HDL-C, mmol/L**	1.27 (1.10–1.47)	1.22 (1.04–1.43)	<0.001	0.17	1.45 (1.26–1.66)	1.39 (1.19–1.58)	<0.001	0.26
**LDL-C, mmol/L**	2.73 (2.32–3.19)	2.80 (2.36–3.27)	<0.001	0.09	2.65 (2.25–3.13)	2.99 (2.50–3.48)	<0.001	0.42
**TG, mmol/L**	1.31 (0.91–1.93)	1.80 (1.25–2.63)	<0.001	0.56	0.90 (0.64–1.29)	1.50 (1.06–2.23)	<0.001	0.90
**FPG, mmol/L**	4.97 (0.60)	5.96 (0.70)	<0.001	1.64	4.87 (0.56)	5.84 (0.72)	<0.001	1.72
**ALT, U/L**	23.00 (16.70–34.00)	28.10 (19.65–43.00)	<0.001	0.37	14.00 (11.00–19.00)	19.50 (15.00–29.00)	<0.001	0.74
**AST, U/L**	23.50 (20.00–28.30)	26.00 (21.40–32.58)	<0.001	0	20.00 (17.10–24.00)	23.05 (20.00–29.00)	<0.001	1
**BUN, mmol/L**	4.80 (4.11–5.60)	4.99 (4.20–5.85)	<0.001	0.14	4.24 (3.58–5.03)	4.66 (3.90–5.51)	<0.001	0.37
**Cr, μmol/L**	80.62 (11.95)	78.49 (13.02)	<0.001	0.22	57.30 (51.90–63.40)	57.85 (51.10–65.00)	<0.001	0.16
**Family history of diabetes**	968 (1.59%)	60 (3.18%)	<0.001		1568 (2.95%)	38 (4.79%)	0.002	
**Smoking status**			<0.001				0.007	
**No**	6,384 (10.49%)	257 (13.61%)			17 (0.03%)	0 (0.00%)		
**Past**	1,270 (2.09%)	45 (2.38%)			10 (0.02%)	1 (0.13%)		
**Current**	13,011 (21.37%)	253 (13.40%)			11,249 (21.18%)	136 (17.15%)		
**Unrecorded**	40,206 (66.05%)	1,333 (70.60%)			41,833 (78.77%)	656 (82.72%)		
**Drinking status**			<0.001				0.055	
**No**	824 (1.35%)	31 (1.64%)			17 (0.03%)	0 (0.00%)		
**Past**	5,102 (8.38%)	113 (5.99%)			306 (0.58%)	3 (0.38%)		
**Current**	14,739 (24.21%)	411 (21.77%)			10,953 (20.62%)	134 (16.90%)		
**Unrecorded**	40,206 (66.05%)	1,333 (70.60%)			41,833 (78.77%)	656 (82.72%)		

DM, diabetes mellitus; other abbreviations as in **Table 1**.

### Association Between Height and DM in Both Sexes

**Table 3** shows the results of a multivariate analysis of the association between height and DM in both sexes. In the unadjusted model, the height of both sexes was negatively correlated with the risk of DM, but after further adjustment for potential confounding factors (models 1–3), the negative correlation still existed in women but disappeared in men. In the model adjusted for age, FPG, family history of DM, TG, DBP, Cr, BMI, SBP, smoking status, drinking status, BUN, LDL-C, and HDL-C (model 3), each increase in 10 cm of female height reduced the risk of DM by 15% (HR per 10 cm increase: 0.85, 95% CI: 0.74–0.98), and the linear trend between height and DM risk disappeared (*P*-trend = 0.6556).

**Table 3:** Cox regression analyses for the association between height and DM in different models in men and women.

	Hazard ratio (95% confidence interval)			
	Unadjusted model	Model 1	Model 2	Model 3
**Men**				
**Height (per 10 cm increase)**	0.70 (0.65, 0.75)	1.05 (0.97, 1.13)	1.04 (0.96, 1.12)	1.06 (0.98, 1.14)
**Height quartile**				
** Q1**	Ref	Ref	Ref	Ref
** Q2**	0.77 (0.61, 0.98)	1.03 (0.81, 1.31)	1.04 (0.82, 1.33)	1.04 (0.82, 1.33)
** Q3**	0.61 (0.48, 0.76)	0.97 (0.77, 1.22)	0.97 (0.77, 1.22)	0.96 (0.76, 1.22)
** Q4**	0.46 (0.37, 0.58)	1.05 (0.83, 1.32)	1.05 (0.83, 1.32)	1.06 (0.83, 1.34)
***P*-trend**	<0.0001	0.5973	0.7153	0.5770
**Women**				
**Height (per 10 cm increase)**	0.46 (0.42, 0.52)	0.85 (0.75, 0.98)	0.86 (0.75, 0.99)	0.85 (0.74, 0.98)
**Height quartile**				
** Q1**	Ref	Ref	Ref	Ref
** Q2**	0.56 (0.48, 0.66)	0.98 (0.83, 1.15)	0.98 (0.83, 1.15)	1.00 (0.84, 1.18)
** Q3**	0.39 (0.30, 0.51)	0.94 (0.72, 1.24)	0.96 (0.73, 1.26)	0.96 (0.72, 1.28)
** Q4**	0.19 (0.07, 0.52)	0.62 (0.23, 1.66)	0.62 (0.23, 1.67)	0.68 (0.25, 1.83)
***P*-trend**	<0.0001	0.4577	0.5124	0.6556

Model 1 adjusted for age, BMI, FPG, and family history of diabetes. Model 2 adjusted for age, BMI, FPG, family history of diabetes, SBP, DBP, smoking status, and drinking status. Model 3 adjusted for age, BMI, FPG, family history of diabetes, SBP, DBP, smoking status, drinking status, BUN, Cr, TG, LDL-C, and HDL-C.

### Height Saturation Effect Points of Women Assessing DM Risk

**Figure 2:**
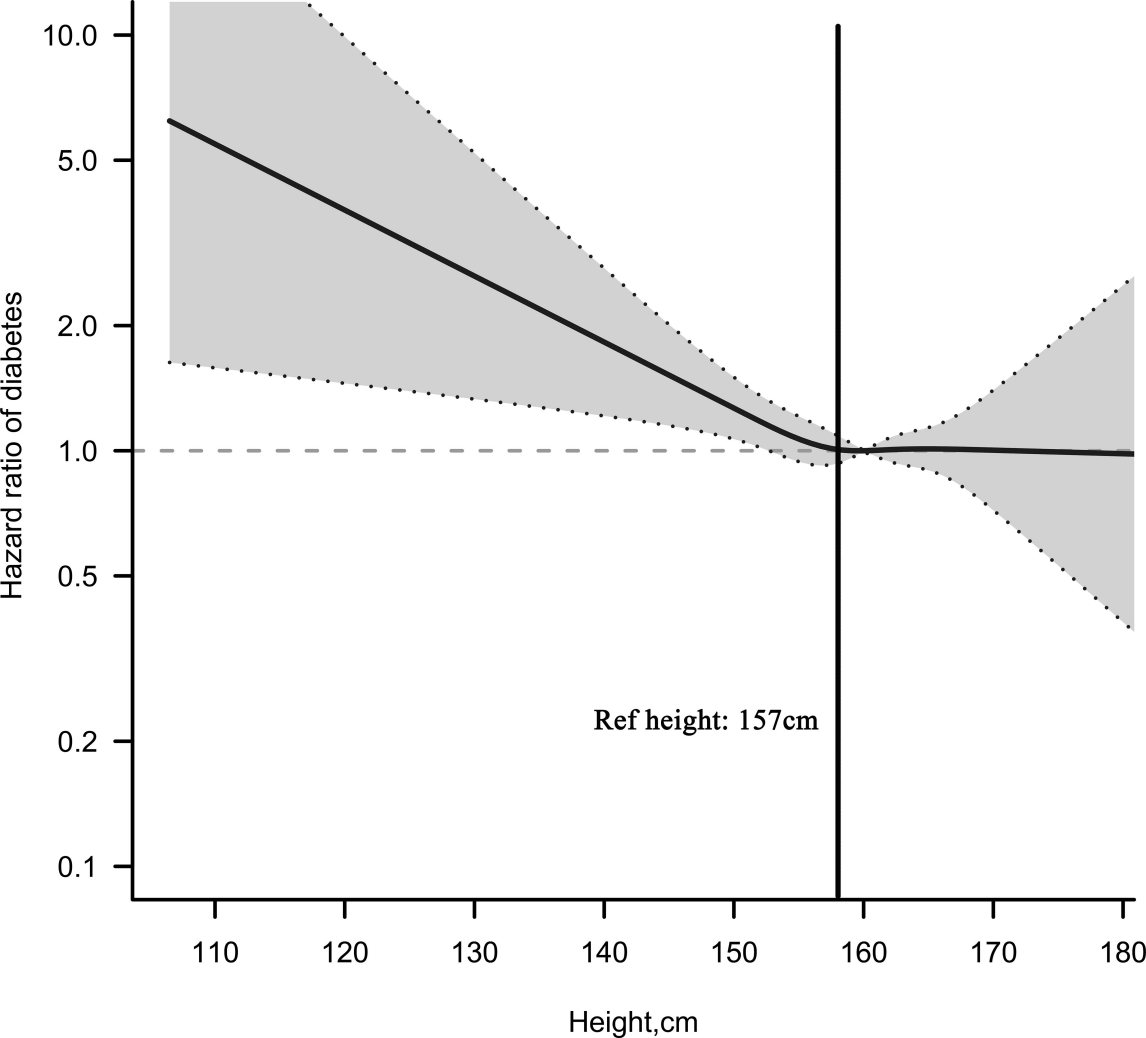
Multivariable adjusted hazard ratios (95% confidence intervals) for the non-linear relationship between height and the risk of diabetes mellitus in Chinese women. Adjusted for age, BMI, FPG, family history of diabetes, SBP, DBP, smoking status, drinking status, BUN, Cr, TG, LDL-C, and HDL-C.

RCS was established to fit the shape of female height and DM risk. As shown in **Figure 2**, there was a negative correlation between female height and DM risk. When the height was about 157 cm, the HR of DM risk was about 1. Additionally, we also calculated the critical value of height for DM risk using a recursive algorithm by a piecewise regression model, and the results showed that the optimal critical value for female height was 157.9 cm. Among people with a height less than 157.9 cm, the risk of DM decreased by 3% for each 1 cm increase in height (HR per 1 cm increase: 0.97, 95% CI: 0.95–0.99), while women who were taller than 157.9 cm had an HR of 1 (**Table 4**). The critical values determined by visual examination and calculated by the recursive algorithm were very close in the current analysis. Therefore, we believe that the female height of 157–158 cm may be a saturation effect point for evaluating the future risk of DM.

**Table 4:** The result of the two-piecewise Cox regression model.

	DM (HR,95%CI)	*P*-value
**Fitting model by two-piecewise Cox regression**		
**The inflection point of height**	157.9 cm	
**≤157.9 cm**	0.97 (0.95, 0.99)	0.0035
**>157.9 cm**	1.00 (0.98, 1.03)	0.7477

Adjusted for age, BMI, FPG, family history of diabetes, SBP, DBP, smoking status, drinking status, BUN, Cr, TG, LDL-C, and HDL-C.

DM, diabetes mellitus; HR, hazard ratios; CI, confidence interval.

### Subgroup Analysis

We also explored the association between height and DM in women of different ages and BMI levels. As shown in **Table 5**, we only observed a negative correlation between height and DM in people older than 60 years old and obese people. However, further interaction tests suggested that there were no significant differences in these findings (*P*-interaction = 0.1508/0.9522).

**Table 5:** Stratified association between height and DM by age and BMI in women.

Subgroup	Unadjusted HR (95% CI)	Adjusted HR (95% CI)	*P-*interaction
**Age (years)**			0.1808
**<45**	0.46 (0.38, 0.57)	0.87 (0.71, 1.06)	
**45–59**	0.68 (0.54, 0.85)	0.88 (0.70, 1.11)	
**≥60**	0.82 (0.69, 0.98)	0.76 (0.63, 0.92)	
**BMI (kg/m^2^)**			0.9522
**<24**	0.46 (0.38, 0.57)	0.86 (0.70, 1.05)	
**≥24, <28**	0.57 (0.47, 0.69)	0.82 (0.66, 1.02)	
**≥28**	0.73 (0.58, 0.91)	0.80 (0.64, 0.90)	

DM, diabetes mellitus; BMI, body mass index; HR, hazard ratios; CI, confidence interval.

## Discussion

The national retrospective cohort study examined the relationship between China’s adult height and new-onset DM. Only female height was found to be significantly negatively associated with DM risk among Chinese adults at a mean follow-up of 3.1 years, an association that remained stable after full adjustment for confounders (HR per 10 cm increase: 0.85, 95% CI: 0.74–0.98). RCS and piecewise regression analysis help us further determine that the height of 157–158 cm may be the critical point for the short stature used by Chinese women to assess the risk of DM.

The relationship between height and DM has always been a controversial topic, and there are significant differences in the results of existing studies based on different places. In a nutshell, the differences are mainly in terms of region and gender. We have made some summary and analysis based on the existing research reports of different regions: 1) Europe: in 1998, a longitudinal cohort study of 11,654 people in Norway revealed for the first time that there was a negative correlation between female height and DM (RR per 5 cm increase: 0.71, 95% CI: 0.58–0.87), but not in men (13). Subsequently, two other longitudinal studies in Europe reported the opposite result: the negative correlation between height and DM was only found in the male population (11, 12). It is worth noting that in the England and German studies, although the association between women’s height and DM was not statistically significant, the lower CI limits of women’s DM risk in these two studies were 0.50 and 0.78, respectively (11, 12). Based on these results, the positive effect of a 22%–56% reduction in DM risk among women cannot be ruled out. 2) North America: Three cross-sectional studies and one longitudinal study have shown that there was no significant correlation between height and DM (16, 26–28), while femur length and leg length-to-height ratio may be the key factors for the assessment of DM in North American population (26, 27). 3) Oceania: A cross-sectional study involving 11,247 Australians showed no relationship between height and blood glucose metabolism (29). 4) Africa: A cross-sectional evidence from Nigeria has shown that there was an association between height and blood glucose levels and glucose tolerance in the African urban population (30). 5) Asia: According to several studies from Asia, there were also some differences between height and DM risk in Asian people, and further distinction may be necessary. i) West Asia: In a survey and analysis in Iran in 2011, only a negative correlation between female height and DM was found after fully adjusting the covariates (14). Although another Iranian study in 2012 found a negative association between height and DM in the whole population, the 2012 study only adjusted for age, gender, and waist circumference and did not adequately account for risk factors (31). ii) South Asia: Evidence from Bangladesh showed a negative association between height and DM risk for both sexes (32), whereas this negative association was observed only among women in the Indian analysis (33). Also, it is worth noting that taller Indian men may increase the risk of DM, which contradicts the conclusions of other studies. iii) East Asia: Several studies on the relationship between height and DM have been conducted in China and South Korea. In the study of Rhee et al. in South Korea, they found that the height of both sexes was positively correlated with the risk of DM (15). However, two surveys in China showed different results from South Korean studies. In a survey and analysis by Conway et al., it was pointed out that there was no significant correlation between height and DM risk in Shanghai population, China, while another data from people in Tianjin Province of China by Li et al. showed that short stature in women was closely related to gestational DM (34, 35). In our current study, we analyzed the national physical examination data of Rich Healthcare Group involving 32 locations in 12 cities in China. The results indicated that height was significantly negatively associated with DM risk in Chinese adults only in women, and no such association was observed in men. Overall, women in Asia, Europe, and Africa were more likely to be negatively associated with DM risk, and short-height women in these regions should pay more attention to the primary prevention of DM, actively understand and learn about DM-related knowledge, and establish a correct concept of eating and exercise. The general recommendations are as follows: set appropriate goals and plans with the help of doctors; reduce the intake of a certain proportion of saturated fatty acids and increase the intake of vegetables, and change lifestyle by increasing the appropriate amount of exercise, losing weight, and reducing exposure to DM-related risk factors.

We have known from some previous studies that there is an inverse relationship between height and the risk of cardiovascular and cerebrovascular disease and relative mortality risk, and the shape of this association is non-linear: the researchers found that when height was within a certain range, the risk of cardiovascular and cerebrovascular diseases and mortality risk decreased significantly (36–38). These findings have greatly helped people to change their awareness of the risk of disease and death. However, at present, the understanding of the height critical point for assessing the risk of DM and the shape of the correlation between them is still very limited. A recent study by Professor Al Ssabbagh from India showed that there seems to be a U-shaped association between women’s height and the risk of DM, in which the height is between 155 and 160 cm and the risk of DM is the lowest (33). In addition, in a recent study on gestational DM by Li et al., they determined that 158 cm may be the critical point of short stature for Tianjin women to assess the risk of gestational DM (35). Among men, an Israeli study showed that people with a height of 170–175 cm are at a critical risk of DM (39). Our current study was based on RCS and piecewise regression analysis to determine that female height between 157 and 158 cm may be the saturation point of DM risk. This finding was similar to the height critical point studied by Li et al. and Al Ssabbagh et al. (33, 35). In view of this result, we call on women with height less than 157–158 cm to pay more attention to early intervention of risk factors for DM.

The relationship between DM and gender was extensively studied in the past. Although there are some differences in the results of local studies, generally speaking, the prevalence of DM in men is higher in the world, but the number of women suffering from DM is higher than that of men (40). This difference is closely related to age. Men are more likely to suffer from DM before puberty, while women are more likely to have DM in old age (40, 41). In the current study, the age of women with DM is higher than that of men (60 vs. 56). From the results of age stratification analysis, we only observe the height-related DM risk of Chinese women in the elderly population. For this particular population, based on some existing research evidence, we speculate that it may be related to the following reasons: it is well known that height decline occurs in both men and women during aging, which may be related to osteoporosis, disc herniation, arthritis, spinal disease, and kyphosis (42, 43). According to the observation of Wang et al., with the increase of age, the bone mineral density reduction rate of Chinese women will be higher than that of men and Caucasians (44). In addition, in women, with the increase of age, body fat deposition increases and fat redistribution becomes more obvious (45, 46), and all these factors significantly increase the risk of DM. In summary, height atrophy and an increase in fat due to some physiological and pathological causes during aging may partly explain the risk of height-related DM.

The pathophysiological mechanism of the association between height and DM is speculative. It has been suggested that height is closely related to heredity and early environmental influence (47), while intrauterine environment, children’s nutrition, growth-related hormone factors, and vitamin D deficiency are considered as potential ways to link peripheral growth impairment with the risk of adult type 2 DM (26, 48, 49). Gender differences in the association between height and DM are not yet clear in the Chinese population. Some studies suggest that early puberty in women causing eventual shorter height may be an important factor (15, 50); however, this statement does not seem to be convincing enough. From the current study, we found that compared with short-height men (Q1), short-height women have some differences with men in the family history of DM (0.85% vs. 2.9%), which further suggests the importance of heredity in this association. Further research is needed to explain this particular gender difference.

Adult height is determined by a combination of roles, mainly divided into proximal and distal roles. At the proximal role, nutrition and early onset of disease play a key role in adult height (51). In general, nutrition is the most important external factor affecting linear growth in height; before the fetus is born, nutritional deficiencies can lead to intrauterine growth retardation, preterm birth, and low birth weight; these consequences are related to height in adulthood (52–54). After the fetus is born, nutrition has a greater impact on growth, among which high-quality protein, mineral trace elements, and vitamin intake are particularly important (52, 55). Studies have shown that supplementation with micronutrients, iodine, iron, folic acid, and calcium during pregnancy can reduce the risk of delivery of a small-for-gestational-age infant. In addition, milk consumption in children after birth is positively correlated with adult height (56, 57). Disease is another key factor in children’s height development, which can affect growth by hindering food intake and the absorption and transport of nutrients to tissues, leading to direct nutrient loss and affecting bone growth or density (51, 52). At the distal role, socioeconomic status plays a key role in adult height (58). Generally speaking, parents’ social class, socioeconomic status, and educational attainment are all important factors in adult height (51); these characteristics directly affect the resources available to the child, the probability of exposure to risk factors, and the health status of the child’s mother. The most immediate challenges include overcrowded growing environments, reduced access to medical assistance, inappropriate feeding practices, poor dietary conditions, and food/liquid contamination, while in socially underdeveloped areas, there are more complex adverse environmental exposures (such as *Aspergillus flavus*), which significantly affect height growth (51, 59). Like height, DM is also caused by a combination of factors. Besides population aging, environmental factors, socioeconomic factors, and lifestyle changes are thought to be responsible for the rapid increase in the incidence of DM globally in recent decades (3–7, 60). Considering that China is still in the stage of economic development, there are still many families in the unfavorable social environment described above. Based on the above analysis, in addition to improving lifestyle, we have several suggestions that need to be mentioned from the Chinese social level: 1) increasing capital investment to improve the unfavorable living environment of residents, 2) guaranteeing the basic living conditions of women and children in poverty-stricken areas of the country, 3) increasing nutritional subsidies for women and children in poverty-stricken areas, 4) improving medical insurance policies and assistance programs for residents in poor areas, 5) strengthening the construction of professional medical teams and improving medical security in poor areas, 6) reinforcing the construction of a grassroots DM control mechanism, 7) establishing a monitoring network system for DM prevention and control, and 8) incorporating the prevention and treatment of chronic diseases into the basic national policy.

This study has several advantages worth mentioning: 1) The participants of the current study are from 32 locations in 12 cities in China. Compared with the previous two similar studies (34, 35), this study will be more representative of the Chinese population. 2) This study adopts a longitudinal design, and for the first time, it is clear that there are gender differences between height and DM risk in the Chinese population. 3) In this study, two different statistical methods were used to determine the saturation effect points for Chinese women to assess the risk of DM, which provided very useful reference materials for the primary prevention of DM.

Some limitations also need to be highlighted: 1) In the current study, DM was diagnosed by FPG and self-reported, and the study population that might meet the diagnostic criteria for postprandial DM could not be identified, thus possibly underestimating the true incidence of DM. 2) As described above, although stratified analysis in the current study found some meaningful results in subgroups, further interaction tests did not show significant differences, which was mainly related to the short follow-up time in the current study, and these subgroup analysis results need to be confirmed in samples with more DM events. 3) The current study did not distinguish the types of DM, which may affect the application of current research results in some special types of DM. 4) Covariates contained in the current research dataset were still limited, and some known risk factors for DM, such as femoral length, waist circumference, and hip circumference, are not included in the dataset, which inevitably leads to some residual confounding (61). 5) Although the participants in the current study come from many different cities in China (Nantong, Wuhan, Hefei, Guangzhou, Chengdu, Changzhou, Shenzhen, Suzhou, Nanjing, Beijing, Shanghai), most of them (10/11) are from southern China, so the results of the current study may be more applicable to people in southern China. The applicability in northern China needs to be explored in further research. 6) Due to the lack of identification information of different locations and physical examination institutions in the current study, it is impossible to evaluate the errors between different physical examination centers and within them, which may affect the results of this study. Further prospective cohort studies are needed to verify the results. 7) The data of the current study were collected from multiple physical examination centers across the country. It is undeniable that there are certain differences in genetic, environmental, nutritional, and physical activities among subjects in different regions, which may affect the interpretation of parameters collected and height saturation point. 8) Although we have excluded subjects with DM at baseline, this study did not evaluate whether non-DM subjects used DM drugs at baseline, which may lead to some errors in the true diagnosis rate of DM.

## Conclusion

In conclusion, the present study confirms that the short stature phenotype of Chinese women significantly increases the risk of DM, and 157–158 cm may be the saturation point of female’s short height for predicting the risk of DM. These findings further clarify the association between height and DM in the Chinese population. These new insights may help develop a more accurate risk prediction model and may allow individuals to change their other behaviors to help reduce the risk of DM.

## Author Contributions

XW and WS designed the study. WS, YH, JY, YW, ZC, JX, and JL analyzed the data. YH, JY, YW, ZC, JX, JL, and XW interpreted the results. WS wrote the first draft of the manuscript. XW contributed to the refinement of the manuscript. All authors contributed to the article and approved the submitted version.

## Conflict of Interest

The authors declare that the research was conducted in the absence of any commercial or financial relationships that could be construed as a potential conflict of interest.

## Publisher’s Note

All claims expressed in this article are solely those of the authors and do not necessarily represent those of their affiliated organizations, or those of the publisher, the editors and the reviewers. Any product that may be evaluated in this article, or claim that may be made by its manufacturer, is not guaranteed or endorsed by the publisher.
